# Factors associated with living will among older persons receiving long-term care in Finland

**DOI:** 10.1177/26323524231212513

**Published:** 2023-11-30

**Authors:** Paula Andreasen, Leena Forma, Ilkka Pietilä

**Affiliations:** University of Helsinki, Helsinki, Finland; Finnish Institute for Health and Welfare, Helsinki, Finland; Faculty of Social Sciences and Gerontology Research Center, Tampere University, Tampere, Finland; Leena Forma is also affiliated with Laurea University of Applied Sciences, Vantaa, Finland; University of Helsinki, Helsinki, Finland

**Keywords:** advance care planning, end-of-life care, living will, older persons

## Abstract

**Background:**

A living will document is known to be an important tool for preparing for future care together with healthcare professionals. A living will supports an older person’s self-determination and autonomy. Only a few studies have approached the underlying factors of a living will document among older long-term care recipients.

**Objectives:**

To explore how common having a living will was among older persons receiving home care or round-the-clock long-term care, as well as to evaluate associations between socio-demographical factors and functional capacity with a living will.

**Design:**

The study population consisted of older persons receiving long-term care in Finland in 2016–2017. Data were collected *via* individual assessments at home or at a care facility. The questions in the assessment covered health, functional capacity, service use, and social support.

**Methods:**

Primary outcome ‘living will’ and associated factors were identified for each person aged 65 or older from RAI-assessment data (Resident Assessment Instrument, RAI). Cross-tabulations with χ²-tests and adjusted binary logistic regression models were performed to evaluate the association between the factors and a living will.

**Results:**

Of the 10,178 participants, 21% had a living will – a greater proportion were female (22%) than male (18%), and a greater proportion of residents in assisted living (25%) and residential care homes (20%) compared with home care residents (15%) had a living will. Female gender (*p* < 0.001), having a proxy decision-maker (*p* = 0.001), increasing age (*p* = 0.003), impairing functional capacity (activities of daily living hierarchy *p* < 0.001, Cognitive Performance Scale *p* < 0.001), instability of health status (Changes in Health, End-Stage Disease and Signs and Symptoms *p* < 0.001), and closeness of death (*p* < 0.001) were significantly associated with a living will among older persons. Extensive differences in results were found between home care clients and clients of round-the-clock long-term care.

**Conclusion:**

Preparedness for the future with a living will varies according to services and on individual level. To reduce inequalities in end-of-life care, actions for advance care planning with appropriate timing are warranted.

## Background

The autonomy of care recipients and the right to self-determination is a central part of patient or client involvement, which has become a prominent policy in Finnish healthcare and social services over the last two decades.^1,2^ Demand for end-of-life care and advance care planning (ACP) are increasing due to the growing numbers of older long-term care or home care residents, who often die in advanced old age.^3^

According to the findings of Fleming *et al.*,^4^ around 75% of older old persons (age range 79–107) eventually die of a final illness that causes pain, depression, pressure sores, or other symptoms and discomfort that increases the need for medical treatment. Finnish national-level guidelines for palliative care recommend ACP for patients with life-limiting illnesses, and the use of a living will has been acknowledged as one of the key components for good quality end-of-life care for older persons.^5,6^

In this study, a living will is defined according to the Finnish national quality recommendation for palliative care and end-of-life care.^5^ A living will is an official document which contains personal statements about the preferred medical treatment and care in a future situation when an older person is no longer capable of expressing themselves and participates in decision-making regarding their own care. In a living will document, an older person may include statements regarding life-prolonging or life-maintaining medical treatments or activities that a person would like to avoid end-of-life in a situation where the medical treatments and activities cause more harm than good. In a living will, an older person may also express other preferences than those related to medical care, such as wishes for their preferred mode for daily routines, nutrition, or social activities. A living will document is valid when an older person loses his or her legal capacity.^7,8^ The term ‘advance directives’ is also used in scientific literature to describe the written statements regarding medical treatments. In the United States, depending on each state’s law, advance directives may include a living will or a proxy directive, a durable power attorney for healthcare.^7^

A living will may be an important part of ACP where a health professional and an older person, together with close ones, plan end-of-life care.^9^ In this study, we use the European Association for Palliative Care white paper definition of ACP:

Advance care planning enables individuals to define goals and preferences for future medical treatment and care, to discuss these goals and preferences with family and health-care providers, and to record and review these preferences if appropriate. (Rietjens *et al*.^9^)

### Good quality palliative care with a living will

According to earlier scientific evidence, advance directives and a living will have positive influences on the quality of an older person’s end-of-life care.^10–12^ For persons living with dementia, results from a systematic review by Wendrich-van Dael *et al*.^13^ showed the ACP document’s impact on a decrease in hospital use and an increase in concordance between actual care received and stated wishes. Collingridge Moore *et al*.’s^14^ study showed that ACP was linked to care home residents’ longer length of stay with fewer hospitalizations. The quality of life of older persons depends not only on how well symptoms are treated and care needs are met, but also whether unnecessary harm due to late transitions can be avoided.^9,15,16^

Older persons’ preferences added to a living will document are often implemented to individual care and service plan by care professionals and further to care practices. National quality recommendation for palliative care, report of Finnish Ministry of Health and Social Welfare, and the recommendations of European Association of Palliative Care^5,9,16–18^ address the meaning of a living will as part of ACP when improving the quality of end-of-life care and service for older persons receiving long-term care.

Despite the recommendations and scientific evidence about the usefulness of a living will, there is not much information available about how socio-demographic and other factors are associated with making written plans regarding end-of-life care among older persons or how common it is to have a living will in different service types. Some arguments have been raised about some professionals’ unwillingness to take up an ACP routine and a living will document due to the distress that planning or an uncertain future causes to the older person. It is also argued that a gap exists between national policies recommending end-of-life care discussions and the implementation of a living will.

In practice, a living will document may not capture the actual needs of the care recipients but simplify and over-structure the discussion about end-of-life care. This may then lead to situations where the benefits for an older person taking part in their own care planning may remain not to be achieved.^3^ Lack of time or infrequent contact between professionals and a care recipient had also been shown as barriers for healthcare professionals to initiate ACP.^19^ For good reason, a person may also change her or his mind about the content of a living will document or even cancel it.^16,20^

### Overview of the earlier scientific evidence about having a living will and underlying factors

Living will prevalence has been evaluated earlier among Finnish older persons in long-term care, but separately in one service type and by using different methodologies. The proportion of living wills among older health service users varies in Finland from 3% at its lowest to 40% at its highest, according to the results from earlier studies by Konttila *et al*.,^21^ Laakkonen,^7^ and Andreasen *et al*.^22^ These three studies’ results are the most relevant to the current study, but their measures are incomparable due to the differences in methodologies used. In two of the studies^21,22^ data were collected retrospectively in long-term care settings, whereas in Laakkonen’s^7^ study data were collected from terminally ill older persons living at home. In addition to Finland, the prevalence of having a living will has been evaluated among nursing home residents in six European countries (Belgium, Finland, Italy, the Netherlands, Poland, and England). The results varied from 0.1% in Italy up to 76.9% in England (Andreasen *et al*.^22^). In Germany, 42–44% of persons aged 60 years or more and 64% of persons aged 85 years or more had written advance directives about preferred medical care.^23^ In Canada, 43% of persons aged 65 years or more had documented advance directives that included statements about preferred medical treatment.^24^

One reason why the results of earlier studies cannot be easily compared to the current study is due to the contents of a Finnish living will document. Most of the previous studies that have evaluated older persons written statements at end-of-life have focused on advance directives. In Finland, a living will not only contains statements about restrictions regarding medical treatments at end-of-life but also personal care wishes. For example, wishes regarding preferred nutrition, social activities, or funeral arrangements can be included in a Finnish living will. Although a living will document is in particular legally valid in terms of preferred medical treatment options when an older person is not capable to make decisions regarding own future care, both statements regarding preferred medical treatment options and care wishes can be included in the document.

Secondly, in the earlier studies, the care settings are limited into one service type; either round-the-clock care or home care. In a previous multi-country study by Andreasen *et al*.^22^ country comparisons between six European countries in nursing home settings were performed regarding living will documents among older persons. However, in this study, we used a large sample covering home care and round-the-clock long-term care recipients for the evaluation of the proportion of those having living will documents in Finland.

Family members and informal caregivers have proven to have an important role in end-of-life care decision-making among older persons receiving long-term care.^25,26^ A person’s interest in signing a living will may also rise due to declining functional capacity.^27,28^ For the older persons with memory disorders, the decline in cognitive status was shown to increase activity in completing a living will document.^29^

Increasing age, education level, the awareness of nearing death, whether a person received specialized palliative care, and the care facility where end-of-life care was received have been found to be significant predictors of documented advance directives with statements about medical treatment or healthcare power attorney in earlier studies.^24,28,30^ Digout *et al*.’s^24^ study showed that older persons who received end-of-life care in a nursing home more likely had documented advance directives compared with persons living at home. Based on the findings from earlier studies, we also know that older persons in assisted living facilities were more likely to have a living will compared with persons living at home,^30,31^ and that an older person’s relation to their health professional and support from relatives have an influence on the willingness to have documented advance directives.^22,23^ In Andreasen’s *et al*.^22^ study, lack of an in-house physician was found to increase the number of written living wills among nursing home residents. When persons aged 85 years or over in Luck *et al*.’s^23^ study were asked about the reason for not having advance directives, the most common answer was that older persons trust their relatives or their physician to make the right decisions on their behalf when necessary.

Although there is scientific evidence about the advance directives and about the factors linked with having a living will, knowledge is scarce and not comparable to the Finnish health and social care setting due to the variation in research designs and study populations of earlier studies. Since there is a rising trend of older persons receiving end-of-life care and dying at home or in long-term care facilities, it would be important to identify the factors associated with having a living will across different long-term care facilities and home care units. With this study, we aim to evaluate the underlying factors of having a living will among older persons in Finnish long-term care in order to better understand the complex situation when an older person is preparing for future care by signing a living will document.

## Research questions

RQ1: How common was having a living will among older persons in home care and round-the-clock long-term care?
RQ2: How were socio-demographic factors and functional capacity associated with having a living will among older persons in home care and round-the-clock long-term care?

## Data and methods

Resident Assessment Instrument (RAI) is a quality improvement and assessment system used widely in Finnish social and healthcare units. In 2021, 43% of older persons aged 75 or over who received regular home care and 52% of older persons aged 75 or over who received regular round-the-clock, long-term care in Finland had their care and service needs assessed by using an RAI questionnaire. In April 2023, use of the RAI system, including use of RAI-assessments, became mandatory in Finland at primary healthcare and social services for older persons.

Data were sourced from the Finnish Institute for Health and Welfare (THL) research data repository. According to the eligible THL RAI data management procedure, a copy of the data from the assessments is stored in THL repository. Pseudonymized RAI research data is administered by THL, and it is available for researchers through an application process. Permission to use RAI research data in this study was received from THL in February 2021.

### Ethical consent

Approval for the use of the RAI research data in this study was obtained from THL ethical committee under the agreement of RAI research permission at THL. Information about the study participants was drawn from register data base. Individual study participants were not contacted in the study.

### Data collection

The original data were collected in 2016–2017 from older persons’ care and service units in Finland at RAI-assessments. The RAI-assessment questionnaire’s items were originally developed by an international research network interRAI and the items were adapted to Finnish older persons’ care. In this study, we used Resident Assessment Instrument for Home Care (RAI-HC) and Resident Assessment Instrument for Long Term Care (RAI-LTC) questionnaires that are developed for persons receiving either home care or round-the-clock long-term care. The RAI-assessment questionnaire consists of over 250 question items. The questions cover most areas of life: demographic background characteristics, health status, service use, social relations, and functional capacity measures. Validity and reliability of RAI-HC and RAI-LTC have been tested in many international studies.^32–34^ The RAI-assessment’s questionnaire is filled in for each individual by a healthcare professional together with the older person. An RAI-assessment is usually made on a regular basis – twice a year or when a person’s health situation or service needs change.

### Participants

Persons aged 65 or more who received regular service in home care, in assisted living or in residential care home in Finland and died in 2017 were included in the study. Such participants received service that was administered by public or private service provider regularly.

Participants were assessed using one of the RAI questionnaires. For the analysis, only one RAI-assessment per person was included. The last RAI-HC or RAI-LTC assessment before death was included for the analyses of each participant. Only the RAI-assessments, in which all sections of the questionnaire were filled in, were included in the analysis.

### Variables

Variables were selected based on their relevance to the research questions and according to the scientific evidence from earlier studies about the research topic.

The primary outcome variable was the older person’s living will that was identified and marked in the RAI-assessment. The variable ‘living will’ had two values (yes/no), and the values were captured in both the RAI-HC and RAI-LTC questionnaires.

Following variables were also based on RAI-HC and RAI-LTC questionnaires.

### Socio-demographic variables

#### Age

Continuous variable age was recoded into a new categorical variable called ‘age group’. Grouping into categories was made based on the observation of the distribution curve of the continuous variable.

#### Gender

Gender is a categorical variable with two categories: male and female.

#### Informal caregiver

Personal assistance in daily activities from an informal caregiver is a question asked only in the RAI-HC assessment. The information was available for home care clients and those persons living in assisted living who answered the RAI-HC questionnaire items. Overall, in assisted living both RAI-HC and RAI-LTC questionnaires were used. Informal caregiver is a two valued variable (yes/no).

#### Mother tongue

Five different language categories based on the answer in the RAI-assessment questionnaire about the mother tongue. Categories were Finnish, Swedish, Sami, Russian, and other. With this question, differences in a living will document between language groups were identified.

#### Service type

The service types are: home care, assisted living, and residential care home. The service types are defined in this study according to the national Care Register for Social Welfare (HILMO) classification. In HILMO, the service types of Finnish healthcare and social service are described in detail.^35^

‘Home care’ refers to social and healthcare services received in an older person’s home. ‘Assisted living’ refers to a care facility or service house where round-the-clock care is provided to residents. Older persons in assisted living are not capable of living independently at home or with the support of home care professionals’ home visits, but in assisted living they receive assistance according to their needs.

‘Residential care home’ refers to an institutional long-term care facility with round-the-clock care for older persons. Persons in residential care homes are not capable of living independently at home or with the support of home care professionals’ home visits, and they receive assistance according to their needs.^35^ According to a previous publication from Ministry of Social Affairs and Health, for residents in round-the-clock care in Finland, the care needs regarding daily activities (activities of daily living hierarchy, ADLh) are more extensive compared with home care residents, and they also need more assistance with their cognitive problems. However, care needs can be as extensive for the residents in assisted living as they are for the residents in residential care home.^36^

#### Proxy decision-maker

The ‘proxy decision-maker’ variable contains information whether an older person has officially appointed another person and given the appointed person the legal right to make decisions regarding the older person’s own health or services.

For the analysis, two questions in the RAI-assessment questionnaires were combined into a new variable called ‘proxy decision-maker’. The RAI-LTC question about whether a person had an official proxy decision-maker appointed and a similar question from the RAI-HC questionnaire were combined. It is worth mentioning that, in the RAI-LTC questionnaire, there was one question about a proxy decision-maker regarding financial issues. The answers to this question were excluded.

### Other background variables

#### Time left

‘Number of days before death’ is a continuous variable and calculated based on the time period between the date of the last RAI-assessment and the date when a person died. For the statistical analysis, the existing continuous variable ‘number of days before death’ was grouped and put into a new categorical variable called ‘time left’. Four categories (<30 days, 30–89 days, 90–180 days, and 180+ days) to death were defined based on the distribution of the original variable and based on the knowledge from previous research findings about last phase of life.^14,37^ Categories were set to support the research focus.

#### Functional capacity and health stability

Functional capacity and health stability were measured using three scales: ADLh, Cognitive Performance Scale (CPS), and Changes in Health, End-Stage Disease and Signs and Symptoms (CHESS), which are constructed from selected items of the RAI-assessment questionnaire.

ADLh is a six-step-scale measure describing a person’s physical capacity performance in four basic activities of everyday life: eating, hygiene, moving, and use of toilet. In this study’s analysis, ADLh categories 1–2 and 3–4 and 5–6 were combined to create the following categories: 0 independent, 1–2 needed some assistance, 3–4 needed extensive assistance, and 5–6 dependent on others.

The CPS (0–6) measures a person’s cognition level and consists of four variables regarding cognitive abilities: daily decision-making, being understood, memory, and consciousness. In this analysis, the CPS categories 1 and 2, 3 and 4 and categories 5 and 6 were combined to create the following categories: 0 intact, 1–2 borderline intact to mild impairment, 3–4 moderate impairment, and 5–6 severe impairment. These categories describe the level of cognition in terms of how independent or dependent on others an older person was.

CHESS is a 0–5 scale describing stability of health. In this study’s analysis, CHESS categories 2–5 were combined to describe an older person’s stable *versus* instable health. CHESS categories were: 0 normal, 1 mild instability, and 2 instable health.

ADLh, CPS, and CHESS are completed by a health professional at the RAI- assessment. More detailed information about the functional capacity measures ADLh and CPS and stability of health measure CHESS is published elsewhere.^32,38,39^

#### Statistical analysis

The frequency tables were calculated to describe the characteristics of the study population by grouping variables (Table 1). Cross tabulation with χ^2^ test was done to describe the proportions of people with a living will document and to find out the statistical significance between the background variables and a living will document (Table 2).

**Table 1.** Characteristics of the study population, in 2016–2017. RAI-HC and RAI-LTC database, THL.

	Total (%)	Home care (%)	Assisted living (%)	Residential care home (%)	Missing values
Total (*n*)	10,178	3631	5027	1520	
Female	64.6	60.1	67.5	65.7	0
Age group					0
65–74	8.6	11.0	7.4	6.4	
75–84	28.9	30.0	27.8	30.2	
85–89	28.5	28.9	28.5	27.4	
90–94	23.6	21.9	25.1	22.6	
95+	10.4	8.1	11.2	13.3	
Official proxy decision-maker					0
Yes	7.9	8.1	9.2	3.1	
Informal caregiver					5954*
Yes	63.9	69.3	31.5	17.9	
Mother tongue					6
Finnish	89.9	90.3	90.1	88.6	
Swedish	9.2	8.7	8.9	11.0	
Sami	0.4	0.1	0.6	0.4	
Russian	0.2	0.2	0.3	0	
Other	0.3	0.7	0	0	
Time left (days)					0
<30	13.4	6.3	16.4	20.4	
30–89	28.4	24.8	31.0	28.4	
90–179	38.0	35.8	39.2	39.5	
180+	20.2	33.1	13.3	11.8	
ADLh					0
0	21.2	56.0	2.0	1.3	
1–2	16.8	23.9	14.1	8.6	
3–4	23.0	15.5	29.6	18.9	
5–6	39.1	4.6	54.2	71.3	
CPS					0
0	11.7	26.4	3.7	2.8	
1–2	30.0	54.8	16.7	15.1	
3–4	30.6	11.7	40.7	42.3	
5–6	27.7	7.1	38.9	39.9	
CHESS**					54
0	22.4	29.1	18.4	19.7	
1	23.2	26.5	21.0	22.6	
2	54.4	44.4	60.6	57.7	

* The question regarding the assistance from close person was only in RAI-HC instrument. Percentage is calculated from the persons who had answered the question.

** Modified CHESS scale.

ADLh, activities of daily living hierarchy; CHESS, Changes in Health, End-Stage Disease and Signs and Symptoms; CPS, Cognitive Performance Scale; RAI, Resident Assessment Instrument; THL, Finnish Institute for Health and Welfare.

**Table 2.** Living will (%) among older persons in home care, assisted living and residential care home, in 2016–2017.

	Total		Home care		Assisted living		Residential care home	
Total *n* (%)	2104 (20.7)		544 (15.0)		1261 (25.1)		299 (19.7)	
Gender		<0.001		0.017		0.018		0.31
Female	21.9		16.1		26.1		20.4	
Male	18.4		13.2		23.0		18.2	
Age group		0.003		0.32		0.023		0.43
65–74	18.6		17.2		21.4		13.3	
75–84	19.7		14.6		23.8		19.4	
85–89	20.4		13.5		25.5		19.9	
90–94	21.1		15.8		24.9		19.8	
95+	24.9		16.7		30		22.8	
Official proxy		0.001		0.97		<0.001		0.93
Yes	25.2		14.9		32.4		19.1	
Informal caregiver		0.087		0.015		0.004		0.62
Yes	18.6		15.9		55.1		60.0	
Mother tongue		0.40		0.29		0.22		0.67
Finnish	20.9		15.3		25.3		19.5	
Swedish	19.2		12.3		23.6		20.4	
Sami	15.0		0		12.5		33.3	
Russian	25.0		0		40		0	
Other	11.1		8.0		50		0	
Time left (days)		<0.001		<0.001		<0.001		0.70
<30	25.5		21.7		28.0		21.6	
30–89	22.6		17.0		26.8		19.3	
90–179	20.5		14.9		24.5		19.7	
180+	15.1		12.3		19.4		17.3	
ADLh		<0.001		<0.001		0.13		0.12
0	13.4		12.7		25.2		30.0	
1–2	17.7		14.3		22.6		13.8	
3–4	24.9		20.4		27.0		22.6	
5–6	23.4		28.1		24.7		19.4	
CPS		<0.001		0.26		0.044		0.99
0	18.4		16.4		28.7		19.0	
1–2	18.4		14.0		28.4		20.1	
3–4	21.8		15.3		23.9		19.3	
5–6	22.9		17.0		24.6		20.0	
CHESS*		<0.001		<0.001		0.37		0.23
0	16.1		9.4		23.8		16.3	
1	19.7		14.4		24.0		21.2	
2	22.9		19.0		25.7		20.3	

* Modified CHESS scale.

ADLh, activities of daily living hierarchy; CHESS, Changes in Health, End-Stage Disease and Signs and Symptoms; CPS, Cognitive Performance Scale.

Adjusted multivariable binary logistic regression models were used to evaluate the associations between explaining factors and a living will document (Table 3). Hosmer–Lemeshow’s goodness-of-fit test was used to assess the model’s adequacy. Interactions between cognition level (CPS) and functional capacity (ADLh), CPS and health stability (CHESS), and CPS and time left were tested. Due to a statistically significant result that was found when testing with CPS and CHESS interaction term, CHESS was not included in the final model.

**Table 3.** Associated factors of living will (1 = yes, 0 = no) among older persons in home care and in round-the-clock long-term care. Multivariate binary logistic regression models, in 2016−2017.

	Total			Home care			Assisted living			Residential care home		
	OR	95% CI	*p*-value	OR	95%CI	*p*-Value	OR	95% CI	*p*-Value	OR	95%CI	*p*-Value
Gender			0.001			0.008			0.044			0.388
Male	1	Ref		1	Ref		1	Ref		1	Ref	
Female	1.20	1.08 −1.34		1.31	1.07 −1.59		1.16	1.00 −1.34		1.13	0.85 −1.50	
Age group												
65−74	1	Ref		1	Ref		1	Ref		1	Ref	
75−84	1.07	0.88 −1.31	0.49	0.89	0.64 −1.22	0.46	1.15	0.87 −1.52	0.33	1.58	0.84 −2.98	0.15
85−89	1.11	0.91 −1.35	0.30	0.81	0.58 −1.13	0.22	1.27	0.96 −1.68	0.09	1.59	0.84 −3.02	0.15
90−94	1.14	0.93 −1.40	0.21	0.99	0.70 −1.39	0.95	1.20	0.90 −1.60	0.22	1.54	0.80 −2.95	0.20
95+	1.35	1.08 −1.70	0.01	0.99	0.65 −1.50	0.95	1.53	1.11 −2.09	0.01	1.84	0.94 −3.64	0.078
Official proxy			<0.001			0.94			<0.001			0.91
No	1	Ref		1	Ref		1	Ref		1	Ref	
Yes	1.37	1.15 −1.62		0.99	0.70 −1.39		1.55	1.26 −1.91		1.05	0.50 −2.20	
Time left (days)												
<30	1.63	1.37 −1.95	<0.001	1.76	1.22 −2.53	0.002	1.68	1.31 −2.15	<0.001	1.29	0.80 −2.09	0.29
30−89	1.46	1.25 −1.70	<0.001	1.40	1.09 −1.79	0.008	1.54	1.23 −1.92	<0.001	1.13	0.71 −1.78	0.62
90−179	1.33	1.15 −1.54	<0.001	1.22	0.97 −1.54	0.092	1.35	1.09 −1.69	0.006	1.14	0.73 −1.77	0.56
180+	1	Ref		1	Ref		1	Ref		1	Ref	
ADLh												
0	1	Ref		1	Ref		1	Ref		1	Ref	
1−2	1.48	1.23 −1.79	0.001	1.31	1.03 −1.68	0.031	0.99	0.60 −1.60	0.95	0.35	0.11 −1.07	0.065
3−4	2.34	1.95 −2.80	<0.001	2.07	1.58 −2.70	<0.001	1.32	0.82 −2.13	0.25	0.63	0.22 −1.81	0.40
5−6	2.11	1.74 −2.54	<0.001	3.20	2.12 −4.84	<0.001	1.14	0.71 −1.85	0.58	0.50	0.18 −1.43	0.20
CPS												
0	1	Ref		1	Ref		1	Ref		1	Ref	
1−2	0.81	0.67 −0.97	0.021	0.75	0.60 −0.94	0.013	0.94	0.66 −1.35	0.75	1.22	0.52 −2.89	0.65
3−4	0.70	0.57 −0.85	<0.001	0.64	0.45 −0.91	0.013	0.71	0.50 −1.00	0.047	1.16	0.50 −2.67	0.73
5−6	0.71	0.57 −0.87	0.001	0.57	0.37 −0.86	0.008	0.73	0.52 −1.04	0.083	1.23	0.53 −2.85	0.64

ADLh, activities of daily living hierarchy; CPS, Cognitive Performance Scale; OR, odds ratio.

Research data material was analyzed using IBM SPSS Statistics (version 28.0).

## Results

The total number of participants was 10,178, of which 65% were female and 63% were 85 years old or older (Table 1). About 56% of the residents in home care were independent in terms of the physical performance of daily activities (measured with ADLh) compared with 2% of the residents in assisted living and 1% in residential care home. More residents (26%) in home care had normal cognition level (measured with CPS) compared with assisted living (4%) and residential care home (3%). In assisted living, older persons had an official proxy decision-maker more often (9% of the residents) compared with home care (8%) and residential care home (3%).

**Figure 1.**
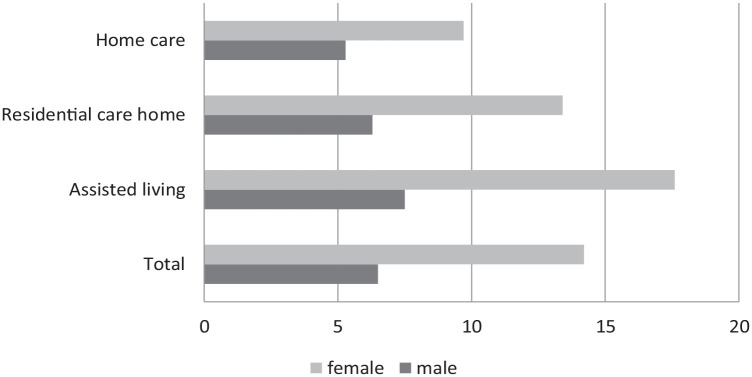
Living will (%) by gender and service type, in 2016–2017.

Of all participants, 21% had a living will registered in RAI-assessment records at the time of the last RAI-assessment which was done on average 118 days before death. A greater proportion of females (22%) than males (18%) had a living will. The occurrence of a living will varied significantly between genders and between different service types (Table 2; Figure 1).

**Figure 2.**
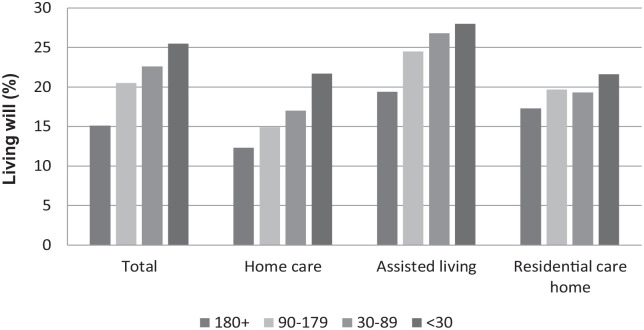
Living will (%) by the time left (in days) and service type, in 2016–2017, RAI-LTC and RAI-HC database, THL. RAI, Resident Assessment Instrument; THL, Finnish Institute for Health and Welfare.

**Figure 3.**
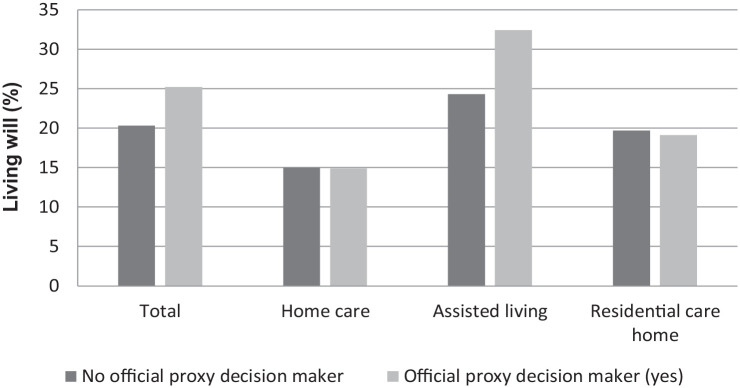
Living will (%) among older persons by proxy decision-maker and service type, in 2016–2017, RAI-LTC and RAI-HC database, THL. RAI, Resident Assessment Instrument; THL, Finnish Institute for Health and Welfare.

**Figure 4.**
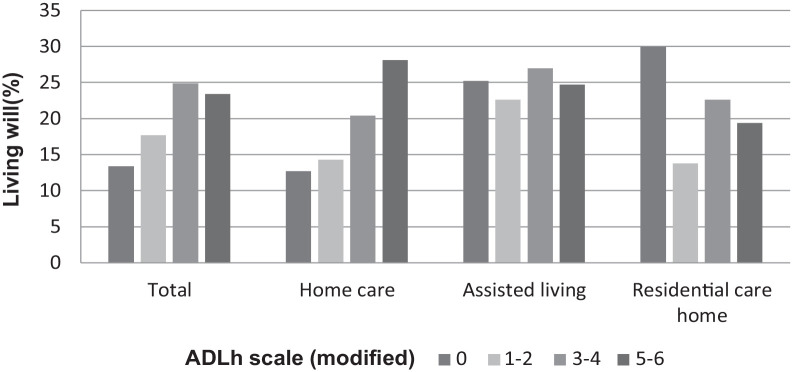
Living will (%) by level of physical performance in daily activities and by service type, in 2016–2017, RAI-LTC and RAI-HC database, THL. RAI, Resident Assessment Instrument; THL, Finnish Institute for Health and Welfare.

Of the different background factors increasing age (*p* = 0.003), female gender (*p* < 0.001), availability of proxy decision-maker (*p* = 0.001), impairing functional capacity (ADLh *p* < 0.001, CPS *p* < 0.001), instability of health status (CHESS *p* < 0.001), and shorter time before death (*p* < 0.001) were found to be significantly associated with living will (Table 2; Figures 2–4). Due to the interaction that was found between the cognition (CPS) and health stability (CHESS), health stability measure (CHESS) was not included in the logistic regression model.

### Multivariate analyses

According to the results of the binary logistic regression model, female participants had signed a living will more likely than male (odds ratio [OR] 1.20 [95% CI: 1.08–1.34]; *p* = 0.001). Significant differences were found between age groups in assisted living, where persons aged 95 years or more had a living will more likely (OR 1.53 [95% CI: 1.11–2.09]) compared with 65- to 74-year-old persons (Table 3).

### Proxy decision-maker

Older persons with a proxy decision-maker were more likely to have a living will than older persons without a proxy (OR 1.37 [95% CI: 1.15–1.62]; *p* < 0.001) (Table 3). Of the different service types, having a proxy was more strongly associated with a living will in assisted living compared with home care and residential care home. Older persons with an official proxy in assisted living were more likely to have a living will (OR 1.55 [95% CI: 1.26–1.91]) compared with persons without a proxy.

### Time before death

At a time of 30 days or fewer before death, a living will had been made more likely compared with the situation of 6 months or more left before death (OR 1.63 [95% CI: 1.37–1.95]) (Table 3). Similarly, at 1–3 months before death (OR 1.46 [95% CI: 1.25–1.70]) and at 3–6 months before death (OR 1.33 [95% CI: 1.15–1.54]) ORs for a living will were higher compared with the situation when there was 6 months or more time left (Table 3).

### Functional capacity

Compared with older persons who were independent in daily activities, a living will was more likely to be made among older persons who needed some assistance, extensive assistance or when a person was dependent on others (Table 3).

Between service types, extensive differences were found regarding the associations between ADLh levels and a living will. In a home care setting, a person’s level of physical functional capacity (ADLh) was associated with having a living will more than in round-the-clock long-term care. Older persons were more likely to have a living will (OR 3.2 [95% CI: 2.12–4.84]) when they were dependent on others or they needed extensive assistance (OR 2.07 [95% CI: 1.58–2.70]) compared with persons who were independent in daily activities. In round-the-clock long-term care settings, ADLh levels were not significantly associated with a living will (Table 3).

A living will was more likely to be made when an older person had normal cognition level (measured with CPS) compared with situations of intact to mild impairment of cognition, moderate impairment, or severe impairment of cognition (Table 3).

In home care, a person’s cognition level (CPS) was associated with having a living will more strongly than in other service types. Persons who had intact to mildly impaired cognition, moderate impairment, or severely impaired cognition were less likely to have a living will compared with persons who had normal cognition level. In contrast to the results in home care, older persons in assisted living with moderate impairment in cognition were less likely to have a living will compared with persons with a normal cognition level (Table 3). In residential care homes, cognition levels were not associated with having a living will.

## Discussion

According to this study’s results, closeness to death, having an official proxy and impairment of functional capacity increased the likelihood of an older person having a living will. Between different service types, extensive differences were found regarding factors significantly associated with a living will.

The results confirmed what was already known from earlier scientific evidence, that impaired functional capacity, age, and gender affect older persons’ likelihood of having a living will.^21,27,28^ However, the impact of the factors was not evaluated earlier in home care and in round-the-clock long-term care separately. In Finland, previous scientific evidence has only focused on one of the service types – distinctly^7,21,22^ and the differences between home care and residential care have not been explored.

The results of this study are in alignment with the findings from earlier studies^28,31^ where, in assisted living, older persons were more likely to have a living will compared with older persons living at home and receiving home care. In assisted living and in residential care homes, the majority of the residents were dependent on others in daily activities and their cognition level had also declined more compared with older persons in home care. With poorer functional capacity and cognition, older persons in round-the-clock long-term care need support from professionals or informal caregivers to discuss future care and to make written plans. This study’s results showed that older persons in assisted living were more likely to have an appointed proxy decision-maker compared with other service types, and they also were more likely to have a living will. Based on the findings from earlier studies, we know that an older person’s relation to their health professional and support from relatives have an impact on having documented advance directives.^22,23^ Many older persons are more independent in their daily activities in a home care setting compared to other long-term care settings, and they might not have regular in-person contact with care professionals who could initiate end-of-life care conversations.

The results of the adjusted multivariate regression model showed that, in home care, all categories of functional capacity in daily activities (ADLh, modified scale) were significantly linked with a living will, whereas in assisted living and in residential care home, no significant associations between physical performance in daily activities and a living will were found (Table 3). Moreover, in a home care setting, all stages of impaired cognition (CPS, modified scale) were found to be significantly linked with a living will. This is in contrast to the fact that in assisted living, only one level – moderately impaired cognition – was found to be significantly associated with a living will (Table 3). Based on the results of this study and proved by earlier studies, losing autonomy seems to be linked to an older person’s activity to complete a written living will.^27–29^ Overall, older persons receiving home care are less dependent on others in daily living than other long-term care residents; however, when their physical or other functional capacity weakens, they actively start planning for their future care needs. However, based on this study’s findings, we cannot make assumptions about the influence of a service type on whether an individual completes a living will. Instead of just focusing on the differences in service types, future research should aim to investigate older persons with different types of care needs in order to establish how good quality end-of-life care with ACP has evolved.

In all service types, closeness of death was associated with having a living will. Preparedness for the future with a living will varies according to the service the older person receives at end-of-life. That is proved by this study and it is aligned with earlier research findings.^24,28,31^ It is necessary to prepare for the future by signing a living will before a person’s physical condition worsens too much. Based on the results of this study, it still remains unclear whether the advanced directives were put in place in a timely manner to support good quality end-of-life care practices. From health professionals’ viewpoint, it is important to pay attention to the older person’s individual needs and preferences at the time when they can be discussed, and future care and service planned without dismissing the support from their nearest ones.^9^

The result showed that appointing a proxy decision-maker increased the individual’s likelihood of having a living will. However, we do not know if the appointed proxy encouraged the older person to sign a living will or if the older person appointed the proxy in order to complete a living will. Similar results were achieved in a study by Choi *et al*.^29^ where it was found that persons with diagnosed Alzheimer’s disease had a written living will and also a durable power of attorney regarding healthcare, but the origins of the actions remained unclear. Nonetheless, in our study, we found that an official proxy was more strongly associated with having a living will in assisted living where residents’ physical functional capacity and cognition level were poorer compared with home care residents.

One limitation of this study is the informal caregiver variables’ insufficient information. Due to the fact that the question about informal caregivers was asked only in one of the questionnaires, it was not possible to make conclusions about the informal caregiver’s impact on a living will in the whole study population. The informal caregiver’s role would have been important to investigate in the study because we know from earlier studies that an informal caregiver has an impact on making a living will, and it is also a well-known fact that – especially for older persons who would like to die at home – the family carer’s assistance is crucial.^3,25,26^

The results gained in this study address the importance of ACP in the care and service units for older persons living at home and in round-the-clock long-term care units. ACP – procedure is highly recommended in national guidelines and care instructions, but – in practice – the level of implementation is fairly unknown. Furthermore, the quality of communication in ACP discussions has often been reported as being poor.^3^ In the light of this study’s results and the earlier scientific evidence, we may suggest that for older persons receiving long-term care in Finland, capabilities with regard to decision-making vary not only on individual level but from service to service. In ACP, where individual care is discussed, not only should an individual’s preferences for end-of-life care and capability to express themselves be considered as important, but it should also be acknowledged that the type of care settings, personnel, and any informal caregiver carry an important role.

The findings of this study increase the understanding of the background characteristics of an older person, which is important to consider when planning equal older persons’ services. The results also highlight the inequalities between home care and round-the-clock long-term care residents in terms of planning for future care with a living will. Particularly in Finland, end-of-life conversations and other ACP practices need to be implemented in both home care and residential care services. In all situations, and despite different backgrounds, the care of an older person should meet the individual’s needs in the best possible way and guarantee good quality of life at its end.

## Declarations

**Ethics approval and consent to participate:** Approval for the use of the RAI research data in this study was obtained from Finnish Institute for Health and Welfare (THL) Ethics Committee under the agreement of RAI research permission at THL. Information about the study participants was drawn from register database. Individual study participants were not contacted in the study and informed consent to participate was not obtained from participants.

**Consent for publication:** Not applicable.

**Availability of data and materials:** Not applicable.
